# Longitudinal transcriptomic dysregulation in the peripheral blood of transgenic Huntington’s disease monkeys

**DOI:** 10.1186/1471-2202-14-88

**Published:** 2013-08-17

**Authors:** Jannet Kocerha, Yuhong Liu, David Willoughby, Kumaravel Chidamparam, Joseph Benito, Kate Nelson, Yan Xu, Tim Chi, Heidi Engelhardt, Sean Moran, Shang-Hsun Yang, Shi-Hua Li, Xiao-Jiang Li, Katherine Larkin, Adam Neumann, Heather Banta, Jin Jing Yang, Anthony WS Chan

**Affiliations:** 1 Department of Neuropharmacology and Neurologic Disease, Yerkes National Primate Research Center, 954 Gatewood Rd., N.E, Atlanta, GA 30329, USA; 2 Department of Human Genetics, Emory University School of Medicine, 615 Michael St. Whitehead Building, Atlanta, GA 30322, USA; 3 Ocean Ridge Biosciences, 10475 Riverside Drive, Suite 1, Palm Beach Gardens, FL 33410, USA; 4 Current address: Department of Chemistry, Georgia Southern University, Statesboro, GA 30458, USA

**Keywords:** Transcriptome, Huntington’s disease, Longitudinal, Monkeys, Blood, mRNA

## Abstract

**Background:**

Huntington’s Disease (HD) is a progressive neurodegenerative disorder caused by an expansion in the polyglutamine (polyQ) region of the Huntingtin (*HTT*) gene. The clinical features of HD are characterized by cognitive, psychological, and motor deficits. Molecular instability, a core component in neurological disease progression, can be comprehensively evaluated through longitudinal transcriptomic profiling. Development of animal models amenable to longitudinal examination enables distinct disease-associated mechanisms to be identified.

**Results:**

Here we report the first longitudinal study of transgenic monkeys with genomic integration of various lengths of the human *HTT* gene and a range of polyQ repeats. With this unique group of transgenic HD nonhuman primates (HD monkeys), we profiled over 47,000 transcripts from peripheral blood collected over a 2 year timespan from HD monkeys and age-matched wild-type control monkeys.

**Conclusions:**

Messenger RNAs with expression patterns which diverged with disease progression in the HD monkeys considerably facilitated our search for transcripts with diagnostic or therapeutic potential in the blood of human HD patients, opening up a new avenue for clinical investigation.

## Background

HD is a dominant genetic disorder caused by pathologic expansion of a polyglutamine (polyQ) repeat in exon one of the IT15 gene encoding *HTT*. The hallmark of HD is neurodegeneration and formation of mutant HTT protein aggregates, predominantly in the striatum and cerebral cortex [1]. The polyQ repeat length influences the age of onset and the overall level of function, and can be correlated with the severity of the disease [2]. However, it has been suggested that CAG length accounted for 40% of the variability in the age of onset [3], thus there are other factors which contribute to the age of onset. The phenotype of the disease is demonstrated through three categories of abnormalities: cognitive, psychological, and motor. Studies have suggested that cognitive deficits often emerge as the first symptom of disease onset, specifically seen in speech delay in juvenile HD patients [4]. Approximately 44% of HD patients experiencing some general cognitive impairment reportedly struggle with memory retrieval [5]. The psychiatric disorders common to HD include aggression, affective disorders, irritability, obsession-like symptoms and behavioral and personality disorders [6].

The average age of onset for HD is 37–38 years [7] and the median life expectancy after disease onset is 16.2 years (with a span of 2–45), with the median being similar regardless of age of onset [8]. Many patients have children before discovering they have the disease. Although there is a single causal genetic mutation in HD with very high penetrance, effective therapeutics to treat disease symptoms remain elusive. Large sequencing or microarray profiling studies of the “transcriptome” (all RNA transcripts present in a species) are beginning to facilitate identification of potential new therapeutic targets in HD; however, extensive research is required as humans generate thousands of transcripts.

There are reports of RNA analysis from a range of biological tissues types as well as from various model systems with HD, including humans and rodents [9-12] Transcript expression has been assessed from distinct brain regions of HD patients [11,12], which is refining our understanding of the molecular alterations with disease progression. Although the brain is a primary pathogenic target in HD, peripheral tissue damage is also prevalent. Studies from peripheral cells which can non-invasively be collected, such as blood, are ideal for longitudinal correlation of transcriptome expression with the stages of disease. Identified candidates from blood could function as putative pathogenic biomarkers or therapeutic targets in pre- and post-symptomatic patients. To date, there are limited reports of RNA analysis from peripheral blood in HD, however, a few laboratories have profiled mRNA alterations from total blood as well as isolated lymphocytes [9,13]. The list of validated targets from the blood cell studies are not expansive and are cross-sectional analyses from only one species, humans.

Here, we propose to elucidate the longitudinal transcriptomic patterns in peripheral blood of transgenic HD monkeys, an animal model which has a long lifespan of many years and are amenable to routine blood collection [14]. Previous reports from our group have shown the HD monkeys uniquely exhibit some symptoms consistent with those exhibited in human patients [14]. We developed a group of HD monkeys which express a fragment of the human *HTT* gene with a range of polyQ repeats greater than the 10–11 polyQ repeats that are normally present in wild-type rhesus macaque. As rhesus macaque endogenously express 10-11Q, the pathogenic threshold of polyQ repeats in monkeys is expected to be lower compared to 35–37 polyQ repeats in healthy human populations [14]. Biological samples are collected on a routine and scheduled basis throughout the lifespan of each animal. We profiled the transcriptome of the monkeys from peripheral blood and identified mRNAs associated with HD during the first two-years of their development, a timespan which encompasses the onset of HD phenotype. We further monitored a select group of candidates, based on their apparent corresponding dysregulation reported in human HD patient blood, for an additional year. Collectively, this study begins to map the molecular dynamics that occur during the course of HD pathogenesis and identify transcriptomic targets with distinct disease trends.

## Results

### Generation of HD transgenic monkeys

**Figure 1:**
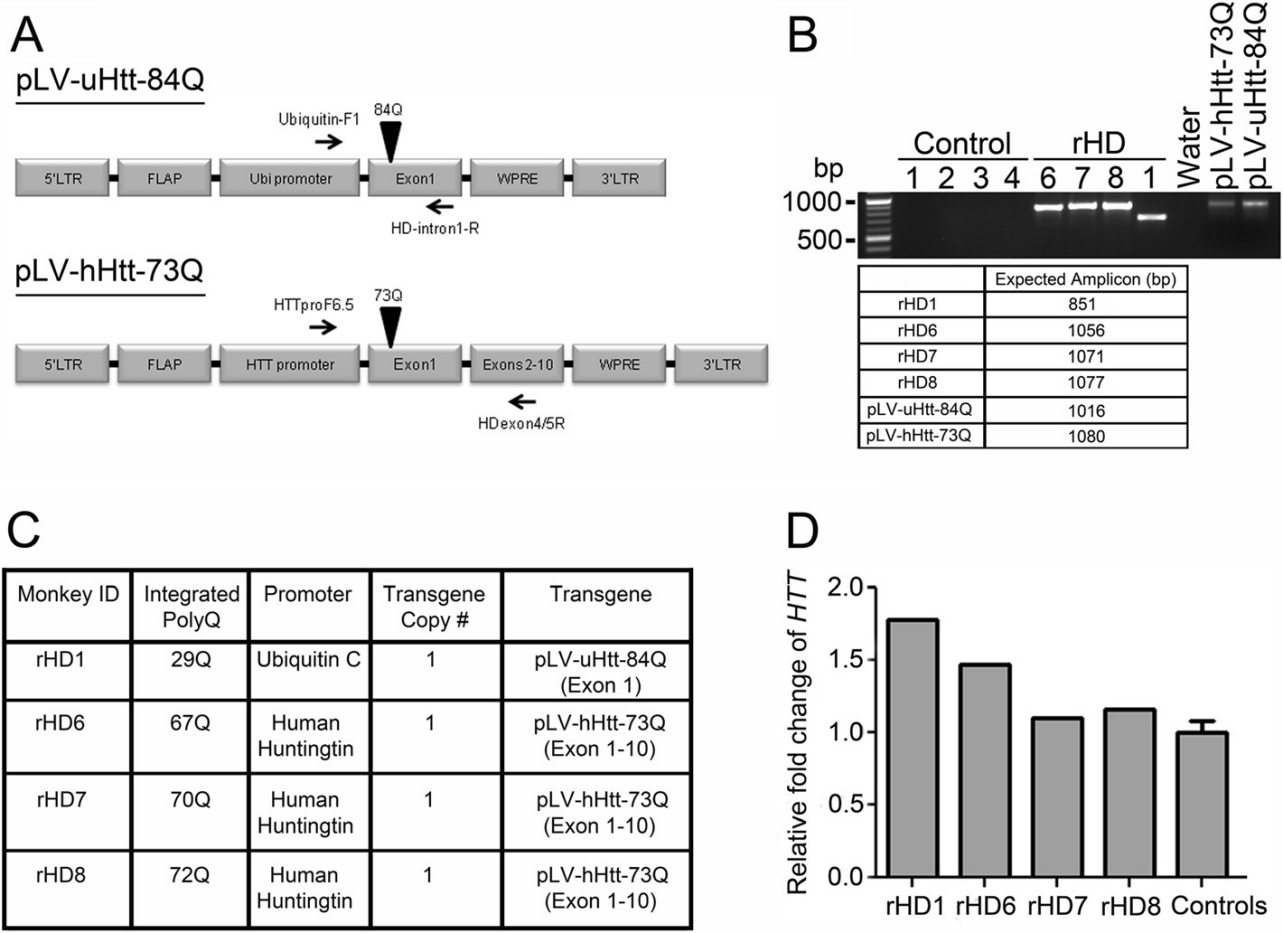
**Molecular characterization of HD transgenic monkey founders. A)** Schematic of lentiviral transgene for the generation of HD monkeys. Primers used to genotype the HD monkeys are depicted in the schematics. **B)** Genotyping of HD monkeys using the primers outlined in panel A. Expected PCR amplicon sizes are detailed in the table. Genotyping was performed using DNA extracted from blood samples of individual founder and control monkeys. **C)** A table describing the promoter, transgene structure, copy number and number of polyQ repeat units integrated into the genome of each HD monkey. All transgene copy number and polyQ analysis was examined in blood samples collected from each monkey. **D)** Fold change of *HTT* mRNA expression by qPCR in all HD monkeys compared to age-matched controls from blood. The qPCR for fold change analysis was set up in technical duplicates for each sample from the individual monkeys. The control group consists of the values for all 4 wild-type monkeys.

Transgenic rhesus macaques were created to overexpress exon 1 (rHD1) [14] or a larger fragment containing exons 1–10 of the human *HTT* gene (rHD6, rHD7 and rHD8), under the control of the human polyubiquitin-C (*UBC*) or human *HTT* promoter respectively, with different number of polyQ repeats (Figure 1A and 1C). rHD1 expresses a 29Q repeat, and rHD6, rHD7, and rHD8 express 67Q, 70Q, and 72Q respectively (Figure 1C). All HD monkeys carry *HTT* with expanded polyQ repeats, based on normal wild-type rhesus macaque carry 10–11 polyQ repeats (Additional file 1), which parallels the genetic anomalies observed in human patients [15-18] (Figure 1A-1C). Integrated polyQ repeat number varies among the HD monkeys, which may be due to the inherent instability of expanded polyQ sequences that has been extensively studied [19,20]. The *UBC* promoter permits more ubiquitous expression of the transgene [14], whereas the *HTT* promoter enables similar tissue-related expression patterns as the endogenous *HTT* transcript [21,22]. Indeed, the endogenous *HTT* is not highly expressed in peripheral blood [9,13], paralleling the lower levels of the transgene *HTT* mRNA levels we observed in rHD6, rHD7 and rHD8 compared to rHD1, which is regulated by the *UBC* promoter (Figure 1D). Based on our preliminary analysis of rHD1 in its first 24 months of life, including imaging findings, cognitive behavioral decline symptoms, and several episodes of seizures at a young age (2 months), this particular animal may parallel human juvenile onset HD (JHD). On the other hand, the other three HD monkeys that carry a larger *HTT* fragment driven by the human HTT promoter seem to have a slower rate in progression without the development of seizures; thus, these HD monkeys may more closely resemble adult onset HD. We developed an HD primate model rating scale (HDPMRS)[14], which is modified from the Unified Huntington's Disease Rating Scale (UHDRS) [23] that is commonly used for monitoring progression of HD in patients. The HDPMRS is primarily focused on monitoring the progression of motor deficit while psychological behaviors such as suicidal thought and speech were excluded. All HD monkeys exhibited progressive scores compared to the control monkeys. At 12 months, there were no apparent differences between the HD and control monkeys by HDPMRS, however, at 24 months (5.25 *vs* 1; p < 0.05, respectively) and 37 months (7 *vs* 1.75; P < 0.05, respectively), the HD monkeys rated significantly above controls (Additional file 2).

### Microarray profiling reveals transcriptomic dysregulation in HD monkeys

To systematically map the transcript expression patterns with HD progression, we interrogated over 47,000 mRNAs in peripheral blood samples collected from the HD monkeys and age-matched controls at 5, 11, 17 and 23 months of age with rhesus macaque Affymetrix arrays. Spiking controls present on the arrays showed very minimal differences across all hybridizations (not shown). We selected 32,617 out of 52,024 Rhesus Affymetrix probe sets on the microarray for further analysis based on a “present” detection call from the Affymetrix MAS5 algorithm in 10% or more of the samples (Additional file 3; currently in progress for GEO submission to NCBI). Two-way ANOVA of log2-transformed probe intensities using a fixed-effect model with disease as a categorical and age as a numeric variable showed 1,521 and 3,341 probes with a significant (p < 0.01 and false discovery rate (FDR) <0.1) disease and age effect, respectively (Additional file 3, Figure 2A and 2B).

**Figure 2:**
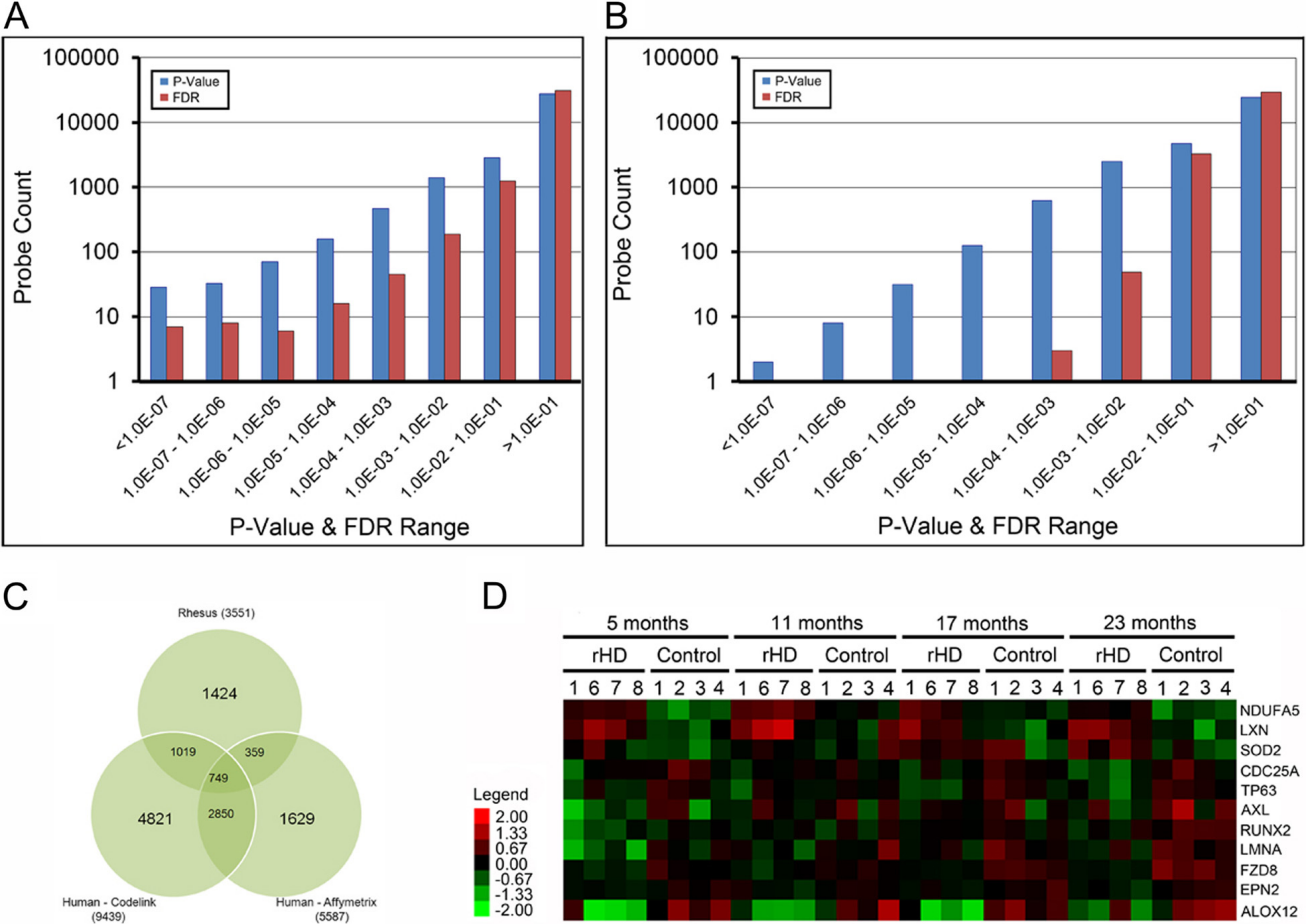
**Genomic profiling of blood from developing HD monkeys.** Total RNA from the peripheral blood was collected at 5, 11, 17, and 23 months and analyzed by Rhesus Macaque Affymetrix arrays. Histograms were generated from the array data. **A)** Probes with P-value and FDR ranges from less than 1.0E-07 to greater than 1.0E-01 obtained from 2-way ANOVA for the effect of disease are plotted. 2-way ANOVA analysis was from data spanning the 5, 11, 17, and 23 month samples. **B)** Probes with P-value and FDR ranges from less than 1.0E-07 to greater than 1.0E-01 obtained from 2-way ANOVA for the effect of age are plotted. **C)** Venn Diagram analysis of overlapping disease-associated mRNAs from the HD monkeys with results from human HD patient blood deposited into the NCBI/GEO database. The human HD blood samples were analyzed independently using two different array platforms (Affymetrix and Codelink) and compared with the rhesus HD-associated mRNAs by Venn Diagram. All analyzed mRNAs have a P value < 0.05. **D)** The expression values (Log2 transformed & normalized microarray probeset intensities) of 11 HD-associated mRNAs identified from the Rhesus profiling were median centered for heatmap analysis. Each column represent a single sample, and each row represents a single mRNA probeset. Green squares represent lower than median levels of mRNA expression; black squares represent median levels of mRNA expression; red squares represent higher than median levels of mRNA expression.

### Significant pathway association with HD dysregulated transcripts

Ingenuity analysis of the mRNAs with a P disease value < 0.001 revealed 54 canonical pathways significantly associated with the dysregulated HD transcripts. Some of those canonical pathways have previously been linked to HD, such as pyruvate metabolism [24], glycolysis/gluconeogenesis [25], NGF signaling [26], and NRF2-mediated Oxidative Stress Response [27] (Additional file 4).

### Parallel disruption of transcript expression in HD monkey and human blood

To identify mRNAs with the strongest potential for association with HD, we compared the rhesus macaque array data with array results deposited into the NCBI GEO database from human blood of control and HD patients [9]. For further stringency in our search for HD-related transcripts, we analyzed two datasets deposited into GEO from the same set of human samples which were probed separately with two independent array platforms (GEO files GDS1331 and GDS1332). Between the two human array datasets (GDS 1331 and 1332), we identified 2850 mRNAs with analogous patterns of disrupted transcript expression (P value < 0.05), and focused on those for further comparison with the rhesus candidates. Notably, 749 of the 2850 human HD transcripts were also altered in our HD monkeys (Figure 2C).

**Figure 3:**
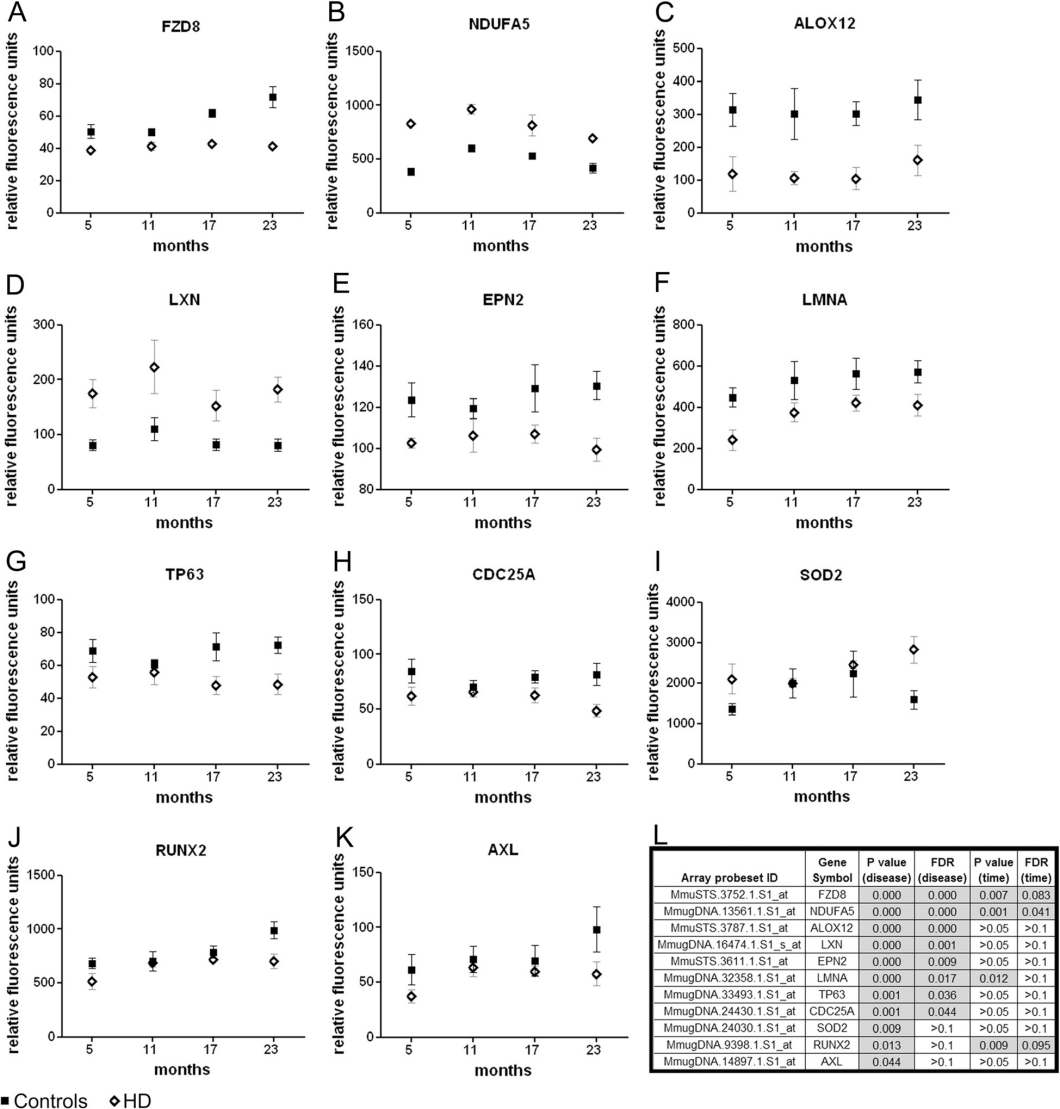
**Longitudinal expression of disease-associated mRNAs from HD monkeys.** The longitudinal trends for selected disease-associated mRNAs from the HD monkeys, with parallel dysregulation in human HD blood, are plotted in panels **A-K**. All data represent raw fluorescence units (log transformed) from the rhesus Affymetrix profiling results of white blood samples collected from 5, 11, 17, and 23 months for each of the 4 control and 4 HD monkeys. The fluorescence values for all 4 controls were analyzed together and the values for all 4 HD monkeys were analyzed together; one blood sample was analyzed per monkey per timepoint. All mRNA candidates have a disease-association P value of < 0.05 by 2-way ANOVA. A table of stats, including P values and FDRs for disease-association and age-effect by Two-Way ANOVA are outlined in panel **L**.

All of the 749 rhesus transcripts with parallel dysregulation in human HD patients were graphed individually to assess the disease-associated trend for each gene and facilitate selection of mRNAs for further investigation. We selected a subset of 11 genes which displayed a significant change in expression between HD and controls across the timepoints analyzed by microarray of 5–23 months by Two-Way ANOVA (P disease < 0.05). Those same candidates were also chosen based on their corresponding dysregulation in studies from human control, presymptomatic and symptomatic HD patients (GEO Database numbers 1331 and 1332) [9], which included; Superoxide dismutase 2 (SOD2), frizzled family receptor 8 (*FZD8),* runt-related transcription factor 2 *(RUNX2),* NADH dehydrogenase 1 alpha subcomplex 5 *(NDUFA5),* cell division cycle 25 homolog A *(CDC25A),* arachidonate 12-lipoxygenase *(ALOX12),* lamin A/C *(LMNA)*, latexin (*LXN)*, AXL receptor tyrosine kinase (*AXL),* epsin 2 *(EPN2)*, and tumor protein p63 (*TP63)* (Figure 2D, Figure 3, Additional file 5, and Additional file 6). Among the eleven candidates with significant changes, four candidates (FZD8, NDUFA5, LMNA, and RUNX2) also displayed an age-effect in the array analysis (Figure 3). Moreover, the 11 selected transcripts collectively encompass a broad range of regulatory and signaling functions, including mitochondrial and immune responses. Candidates, including *NDUFA5, ALOX12, LXN, EPN2, LMNA,* FZD8, and SOD2 began to display altered expression with HD early in development, by 5 months of age (Figure 3). Notably, those same candidates also exhibited an apparent trend for altered expression in presymptomatic human HD patients, suggesting those transcripts may play a role in the earlier stages of HD pathogenesis (Additional file 5). The human study, however, is a cross-sectional analysis, therefore, a full longitudinal study will help further define the progression of molecular changes.

### Longitudinal transcriptomic analysis in HD monkeys by real time PCR (qPCR)

**Figure 4:**
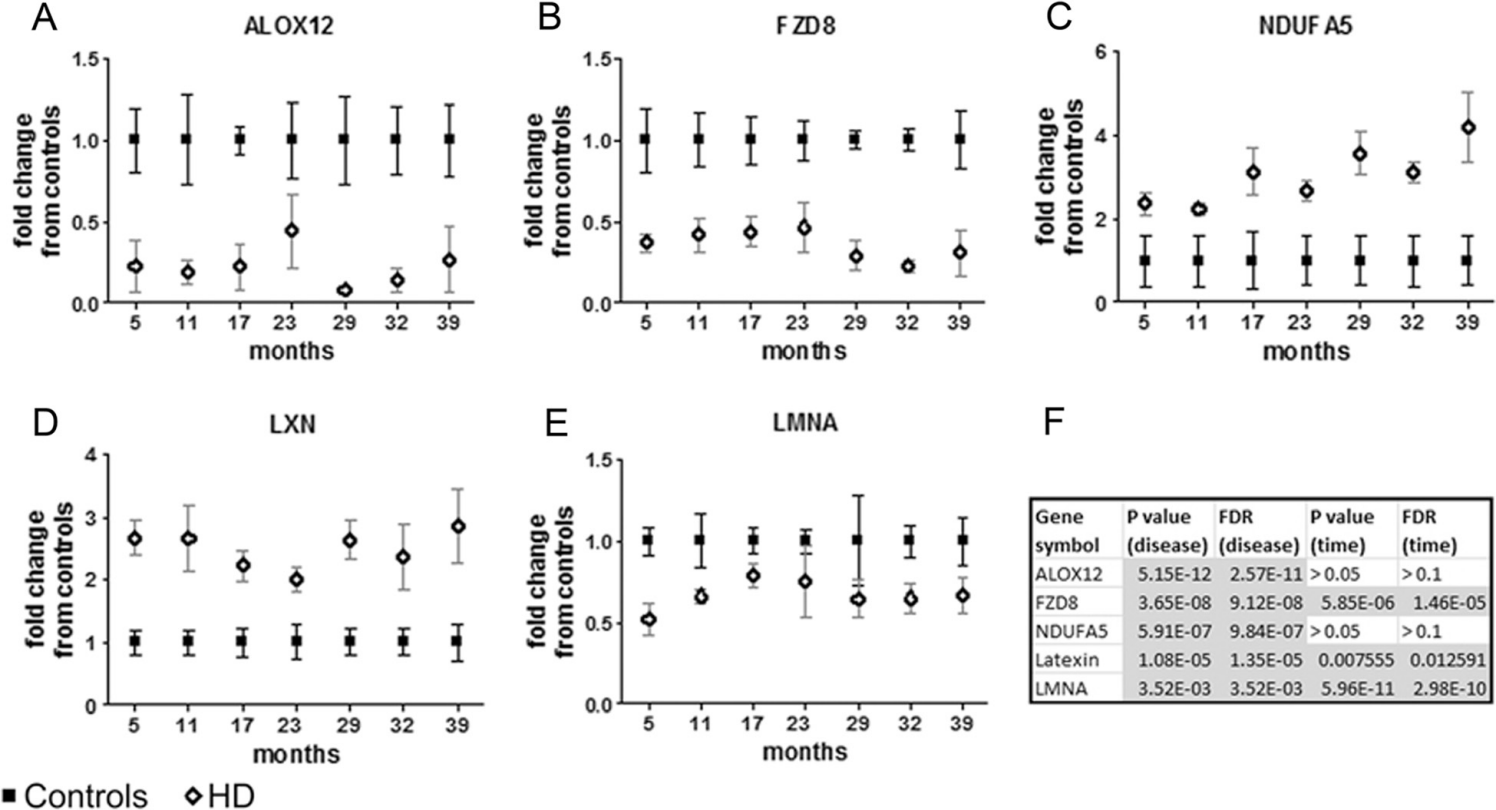
**Extended examination of HD-associated mRNAs by qPCR from blood of HD monkeys.** Five of the selected HD-associated mRNA transcripts with strong divergent trends (*FZD8*, *NDUFA5*, *ALOX12*, *LMNA*, *LXN; *panels **A***-***E**) were further subjected to evaluation by qPCR at extended developmental timespans of 29, 32, and 39 months of age from all control and HD monkeys. Additionally, the 5, 11, 17, and 23 month results timepoints for those same 5 candidates, initially examined by microarray, were also validated by qPCR. White blood cells were collected at each timepoint, followed by RNA extraction and real-time PCR (qPCR) analysis with gene specific Taqman assays (Applied Biosystems). The samples for qPCR were repeated in duplicates for each monkey. The values for all 4 controls were analyzed together and the values for all 4 HD monkeys were analyzed together; one blood sample was analyzed per monkey per timepoint. All qPCR results were normalized with the geometric mean of two endogenous controls, beta-actin (*ACTB*) and glyceraldehyde-3-phosphate dehydrogenase (*GAPDH*). A table of stats, including P values and FDRs for disease-association and age-effect by Two-Way ANOVA with repeated measures are outlined in panel **F**.

To further examine transcriptome changes in the HD monkeys, we measured all 11 mRNAs at extended timepoints of 29, 32, and 39 months to validate the continuous longitudinal changes of the candidates (Figure 4, Additional file 7). Indeed, 5 genes *(NDUFA5*, *FZD8*, *ALOX12*, *LXN*, and *LMNA*) showed longitudinal statistical dysregulation by two-way ANOVA from 5–39 months (Figure 4F). Additionally, all 5 genes appeared to exhibit a trend of altered expression at an early age, at least by 5 months; furthermore, *NDUFA5* showed a trend of progressive dysregulation with age. Statistical comparison of the slopes for the HD and control monkeys for *NDUFA5* from 5 to 39 months revealed significantly different trajectories (P value < 0.05 by Analysis of Covariance).

## Discussion

Here we report the first longitudinal transcriptomic profiling study from an HD monkey model. HD is a highly progressive disorder [28], therefore, identifying patterns in mRNA expression which correspond with disease advancement will likely facilitate the development of novel therapeutic strategies [29]. Distinct alterations in the HD transcriptome were discovered during the first two years of examination from peripheral blood. Some of the identified genes in this study, such as *NDUFA5*, are part of the canonical HD signaling pathway (KEGG pathway for Huntington’s disease) [30], suggesting that alterations in genes commonly associated with *HTT*-mediated pathogenesis can be evaluated in peripheral blood. Moreover, novel HD-associated mRNAs, such as *LXN* or *FZD8*, were also identified through the correlation of HD monkey and human profiles. Interestingly, *LXN* was previously shown to be an important regulator of hematopoiesis [31-33], while deficiencies in peripheral blood functions of HD patients have been widely reported, including immune responses [34-36]. Furthermore, the Wnt signaling cascade, which FZD8 is a component of, is increasingly being reported as an important regulator of the cellular responses to mHTT in HD [37-39]. Transcriptome changes in human HD patients can be significantly influenced by other co-existing pathology unrelated to HD, which arguably would be a less pervasive influence in HD monkeys. It would be anticipated, however, that the primary molecular targets of *mHTT* would be altered in both humans and monkeys, in spite of other physiological or genetic factors which might be present and non-specific to HD. Overall, our data suggests the HD monkey model may help to uncover primate-associated transcriptomic changes due to *mHTT*.

All five candidates we validated with disease associated trends up to 39 months of age in the HD monkeys (*FZD8*, *LMNA*, *LXN*, *NDUFA5*, *and ALOX12*) were dysregulated at a very young age, presumbly in the earlier stages of life based on HDPMRS, while other ongoing clinical evaluations including cognitive behavior and imaging studies will further define their phenotypes. Genes with disrupted expression prior to onset of cognitive and behavioral deficits could potentially be well suited as therapeutic targets aimed to prevent development of disease symptoms. Furthermore, *NDUFA5* appeared to also show a longitudinal escalation in transcript dysregulation with time and disease, suggesting it may also serve as a blood biomarker for HD progression. Interestingly, very recent reports indicate the mitochondrial respiratory chain, which *NDUFA5* is part of, was functionally impaired in platelets from pre-symptomatic and symptomatic HD patients [40]. The upregulation of NDUFA5 mRNA expression in the peripheral blood of humans and monkeys with HD may represent a potential compensatory mechanism as pathology develops. Although this study focused on samples with a mixed population of WBC in both the monkey and human analysis, a future point of interest could entail examining mRNA expression patterns in distinct classes of WBC as well [13]. A cross-sectional study which profiled mRNAs from lymphocytes has been reported (GEO file GDS2887) [13], future longitudinal studies in HD primate models focused on specific WBC cell types could help to further define the location of the molecular changes.

Collectively, our data indicate that HD monkeys can be a useful resource for uncovering transcriptomic targets with correlation to disease progression. Furthermore, longitudinal examination of disease-associated candidates can be monitored from the blood of HD monkeys and correlated with human HD patients for identification of causative pathogenic pathways. Our results reporting consistent molecular changes between HD monkeys and human patients suggest the HD monkeys may be a potential model for future pre-clinical trials. Additionally, the HD monkeys are not exposed to pharmacological agents, thus preventing a drug-based bias in the longitudinal studies.

As the HD monkeys further develop, future studies will focus on extensive molecular mapping encompassing early to late stages of HD progression and define which candidates may be optimal as biomarkers or as therapeutic targets. Upcoming studies will also be able to correlate transcriptomic changes with primate-specific HD behavioral phenotypes, as well as other clinical measurements [14]. Additionally, although the 11 candidates identified in this paper were significantly changed with HD by microarray and extended longitudinal analysis by qPCR, there is a possibility for gender bias due to small sample size. In the current study, there are 4 male HD monkeys and for the controls, there are 2 females and 2 males; thus, studies with future generations of HD monkeys can further examine any influence of gender or other factors on the candidate gene expression. Recently, we reported germline transmission of the human *HTT* transgene along with the presence of pathogenic mHTT aggregation in neurons derived from embryonic stem (ES) cells of rHD1 fertilized embryo [41], setting the stage for creation of the next generation of HD monkeys and corresponding studies.

## Conclusions

We anticipate that on-going longitudinal studies in the current group of HD monkeys as well as future generations will lead to a defined list of transcripts strongly correlated with the progressive pathogenic stages in HD. Future generations of HD monkeys can be engaged in testing of novel pharmacological treatments, offering new approaches to investigate HD.

## Methods

### Transgenic HD monkeys

We developed four transgenic HD monkeys, born and raised at the Yerkes National Primate Research Center of Emory University strictly following the IACUC protocols for animal care. The control group was composed of two females and two males, and the HD transgenic group consisted of 4 males generated through a lentiviral-mediated method as described by Yang and colleagues [14]. In brief, lentiviruses carrying Exon 1 of the human *HTT* gene (NCBI Reference Sequence: NM_002111.6) containing 84 CAGs under the control of ubiquitin promoter (LV-uHtt-84Q) and lentiviruses carrying Exon 1–10 of the human *HTT* gene containing 73Q under the control of human *HTT* promoter (LV-hHtt-73Q) were used to infect metaphase II arrested rhesus macaque oocytes followed by fertilization and embryo transfer into surrogate females. The number of pathogenic polyQ repeats, copy numbers of transgene per cell and general genomic structure of transgene for each monkey are outlined in Figure 1.

### HD rating

Huntington’s disease primate model rating scale (HDPMRS) is modified from the Unified Huntington’s Disease Rating Scale (UHDRS) [23] to evaluate the presence and severity of motor symptoms that classically accompany the disease (Additional file 8). The first section of this battery is specific to the subject’s motor ability and the second section serves as a functional assessment. The HDPMRS is specifically to address motor symptoms of HD monkeys. Video recording was performed once each month for 30 minutes in a cage inside the room where the animals were housed. Scoring of pre-recorded video was performed by a neurologist specialized in movement disorders who did not directly work with the animals, while videos clips were only provided for scoring purpose. T-Test was used to determine significant differences between HD and control animals.

### Blood collection and isolation of white blood cells (WBC)

Blood was collected and processed from all HD and control monkeys at 6 month intervals of 5, 11, 17, and 23 months of age. In brief, the isolation of WBC utilized gradient separation with lymphocyte separation media (Lonza). The WBC containing layer from whole blood collected in heparinized tubes was diluted with phosphate buffered saline (PBS) and pipetted on top of the lymphocyte separation medium followed by centrifugation for 15 minutes at 20°C. After centrifugation, the interphase containing the mononuclear WBC are removed and washed twice with PBS prior to storage of pellet at −80°C.

### Genotyping and polyQ analysis of HD monkeys from WBC

Genomic DNA (gDNA) was isolated from blood collected from all HD monkeys for genotyping and sequence analysis. gDNA was extracted using the Promega Wizard kit and quality was assessed by gel electrophoresis. Primers for PCR amplification extended from the promoter to beyond the polyQ region for all HD monkey transgene analysis. Primer sequences for HD monkey rHD1, generated with pLV-uHtt-84Q, were Ubiquitin-F1 5′- GAG GCG TCA GTT TCT TTG GTC -3′ and HDintron1-R 5′- GCT GGG TCA CTC TGT CTC TG -3′. Primer sequences for HD monkeys (rHD6, rHD7, and rHD8) generated with pLV-hHtt-73Q, were: HTTproF6.5 5′- GTT CTG CTT TTA CCT GCG GC -3′ and HDproexon4/5R 5′- CCG AGG GGC ACC ATT CTT TTT -3′. All reactions were carried out with the annealing temperature of 62°C for 35 cycles in the BioRad iCycler. Amplified PCR products were cloned into the pGEMT-easy vector (Promega) and sequenced by Genewiz.

To obtain the relative copy number of mutant *HTT* transgene in HD monkeys, gDNA was analyzed by qPCR using two independent means of normalization for result confirmation. First, using the HTTexon1 Taqman primers listed in Additional file 9, which recognizes both the endogenous and transgene *HTT*, all reactions were run in a final 1× volume of Fast Advanced Master Mix (Applied Biosystems) and normalized to the geometric mean of GAPDH and β-actin. The normalized Ct values of each HD monkey were then compared to the average of the normalized Ct values for the control monkeys. In an independent experiment, we also normalized Ct values obtained with the HTTexon1 Taqman primers with Ct values using a Taqman assay designed to only recognize the endogenous rhesus *HTT* gene (HTTexon26). The exon1 Ct values of each HD monkey were normalized to their exon26 Ct values and compared with the average of the control monkeys.

For qPCR analysis of *HTT* mRNA expression from blood, 750 ng of total RNA was reverse transcribed to cDNA with the High Capacity Reverse Transcription Kit (Applied Biosystems). Quantitation of *HTT* transcript levels were evaluated by qPCR using a custom-designed gene-specific Taqman assay (Applied Biosystems) and reactions run on the BioRad CFX96 cycler (HTTexon1 Taqman assay in Additional file 9). All data were normalized with the geometric mean of GAPDH and β-actin with Taqman assays.

### RNA isolation from WBC

For total RNA isolation from the WBC pellets collected from the control and HD monkeys, we used a Trizol-based protocol. Briefly, each WBC pellet was homogenized in 500 μl of Trizol (Invitrogen). A phenol-chloroform extraction of the RNA was done by addition of 100 μl of chloroform to the Trizol homogenates followed by centrifugation at 12,000 × g for 10 minutes at 4°C. The aqueous layer was removed for RNA precipitation overnight with isopropanol and 40 μg of glycogen (Invitrogen) at −20°C. The precipitated RNA was pelleted at 12,000 × g for 30 minutes at 4°C. All RNA pellets were washed twice with 75% ethanol and then subsequently dissolved in water (RNase/DNase free).

### Affymetrix array profiling

In a longitudinal gene expression study, RNA from WBCs of HD and control monkeys collected at four age-points (5, 11, 17, and 23 months) were analyzed by using Affymetrix Rhesus Macaque Genome arrays. All microarrays and 2-way ANOVA statistical analysis was done at Ocean Ridge Biosciences.

Biotin-labeled cRNA samples prepared from total RNA using Affymetrix 3′ IVT Express Kit (PN 901229) were hybridized onto the Rhesus Macaque Genome arrays (PN 900655) for 16–18 hours. Scanned images of the arrays were analyzed using RMA algorithm to export the data CHP files using the Affymetrix Expression Console software, version 1.1. Statistical analysis (2-way ANOVA) was performed by dividing the samples into two treatment groups (HD, CTRL) and four age-points (5, 11, 17, 23 months) using BRB-ArrayTools, version 4.1. Detailed descriptions for the Affymetrix array methods and analysis are further outlined in Additional file 10.

### GEO database analysis for human blood

To compare the mRNA profile from the HD monkeys with those from human HD patients, we analyzed genome array results from the blood of human subjects deposited into the NCBI GEO database. The studies consisted of WBC isolated from 14 controls and 12 symptomatic HD patients and analyzed by both Affymetrix and Codelink arrays (Geo files GDS1331 and GDS1332 respectively). We calculated the fold change and P values for each probeset from both the Affymetrix and Codelink gene expression profiling. For added stringency, we identified mRNA candidates with similar dysregulation patterns (P value ≤ 0.05) between the two sets of array data. The genes with similar disruption between the two human studies were then compared with the significant probesets (P value ≤ 0.05 by two-way ANOVA) identified in our array profiling results from the HD monkeys.

### mRNA candidate validation

For validation of selected mRNA candidates, 200 ng of total RNA for each sample was reverse-transcribed to cDNA using the High Capacity RNA to cDNA kit (Life Technologies), following the manufacturer’s protocol. For qPCR, 1 μl of cDNA was primed with custom-designed Taqman assays from Applied Biosystems for the target mRNA in a final reaction volume of 1× Taqman Universal PCR Master Mix (Life Technologies). Primer sequences for all Taqman assay primers and probes are listed in Additional file 9.

## Competing interests

The authors have no commercial association that might create a conflict of interest in connection with the submitted manuscript.

## Authors’ contributions

JK-experimental design, sample preparation, data-analysis, wrote the manuscript; YL-data analysis; KC, JB, DW-array processing and data analysis; YX-HDPMRS scoring; KL, TC, HE, SM, KL, AN, HB-animal care, sample collection and processing; SHY-construct design and generation of HD monkeys; SHL and XJL-construct design; JJY-sample preparation and processing; AWSC-construct design, generation of HD monkeys, experimental design, wrote and approved the manuscript. All authors read and approved the final manuscript.

## Supplementary Material

Additional file 1 **Wild-type rhesus macaque polyQ sequence.** The polyQ sequence from the UCSC genome database was further confirmed and aligned with polyQ sequence from a wild-type rhesus housed at the Yerkes National Primate Center. Click here for file

Additional file 2 **HD rating scores.** HD rating scores by HDPMRS at between 12 and 37 months for all HD and control monkeys. Click here for file

Additional file 3 **Microarray data.** Raw fluorescence values for each sample at each timepoint from the Affymetrix Rhesus Macaque array analysis. Click here for file

Additional file 4 **Ingenuity analysis of HD monkey profiling results.** The Ingenuity pathway program was used to identify signaling pathways (P value < 0.05) associated with HD mRNA transcripts dysregulated with a P value < 0.001 in the Affymetrix profiling. Click here for file

Additional file 5 **Human HD candidate graphs.** Eleven selected transcripts from human HD blood array data (obtained from the GEO database) were graphed from control, pre-symptomatic, and post-symptomatic patient samples. Click here for file

Additional file 6 **Human HD array probeset information.** The human array probeset information for the selected HD-associated candidates. Click here for file

Additional file 7 **qPCR expression analysis of selected HD mRNA candidates at extended timepoints.** The mRNA expression of eleven candidates with HD association in monkey and human blood were analyzed at additional timepoints of 29, 32, and 39 months. Click here for file

Additional file 8 **HD Rating Scale.** Parameters measured in the HD primate rating scale. Click here for file

Additional file 9 **Rhesus Taqman assay information.** All forward, reverse, and probe sequences for rhesus Taqman assays engaged for this manuscript. Click here for file

Additional file 10 Microarray methods. Additional information regarding the Rhesus Macaque Affymetrix profiling are outlined. Click here for file
