# Supplementary material for: Longitudinal transcriptomic dysregulation in the peripheral blood of transgenic Huntington’s disease monkeys
Source: BMC Neurosci. 2013 Aug 17;14:88. doi: 10.1186/1471-2202-14-88 (PMC3751855; doi:10.1186/1471-2202-14-88)
Supplement: Additional file 1 — Wild-type rhesus macaque polyQ sequence. The polyQ sequence from the UCSC genome database was further confirmed and aligned with polyQ sequence from a wild-type rhesus housed at the Yerkes National Primate Center. [file 1471-2202-14-88-S1.doc]

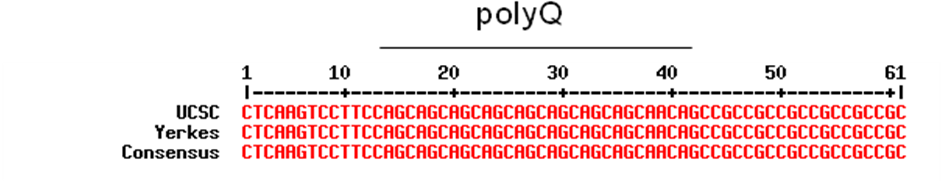


**Additional file 1. PolyQ sequence from Wild-type rhesus macaque**. The polyQ sequence from wild-type rhesus macaque obtained from the University of California at Santa Cruz (UCSC) genome database was aligned with sequence we generated from a wild-type rhesus at the Yerkes National Primate Center. Both the UCSC sequence and Yerkes rhesus sequence revealed a wild-type polyQ length of 10Q.
