# Supplementary material for: Longitudinal transcriptomic dysregulation in the peripheral blood of transgenic Huntington’s disease monkeys
Source: BMC Neurosci. 2013 Aug 17;14:88. doi: 10.1186/1471-2202-14-88 (PMC3751855; doi:10.1186/1471-2202-14-88)
Supplement: Additional file 2 — HD rating scores. HD rating scores by HDPMRS at between 12 and 37 months for all HD and control monkeys. [file 1471-2202-14-88-S2.doc]

Additional file 2. HD rating scores by HDPMRS

| **Age** | **HD1** | **HD6** | **HD7** | **HD8** | **C1** | **C3** | **C4** | **C2** |
| --- | --- | --- | --- | --- | --- | --- | --- | --- |
| 12 months | ~ 0 | ~ 0 | ~ 0 | ~ 0 | ~ 0 | ~ 0 | ~ 0 | ~ 0 |
| 24 -25 months | 6 | 7 ** | 3 ** | 5 | 0 | 2 | 2 | 0 |
| 37 months | 11 | 7 | 4 | 6 | 2 | 0 | 3 | 2 |
|  |  |  |  |  |  |  |  |  |
|  | **HD6 and HD7 rated at 25 months instead of 24 | | | | | | |  |
