# Supplementary material for: Longitudinal transcriptomic dysregulation in the peripheral blood of transgenic Huntington’s disease monkeys
Source: BMC Neurosci. 2013 Aug 17;14:88. doi: 10.1186/1471-2202-14-88 (PMC3751855; doi:10.1186/1471-2202-14-88)
Supplement: Additional file 5 — Human HD candidate graphs. Eleven selected transcripts from human HD blood array data (obtained from the GEO database) were graphed from control, pre-symptomatic, and post-symptomatic patient samples. [file 1471-2202-14-88-S5.doc]

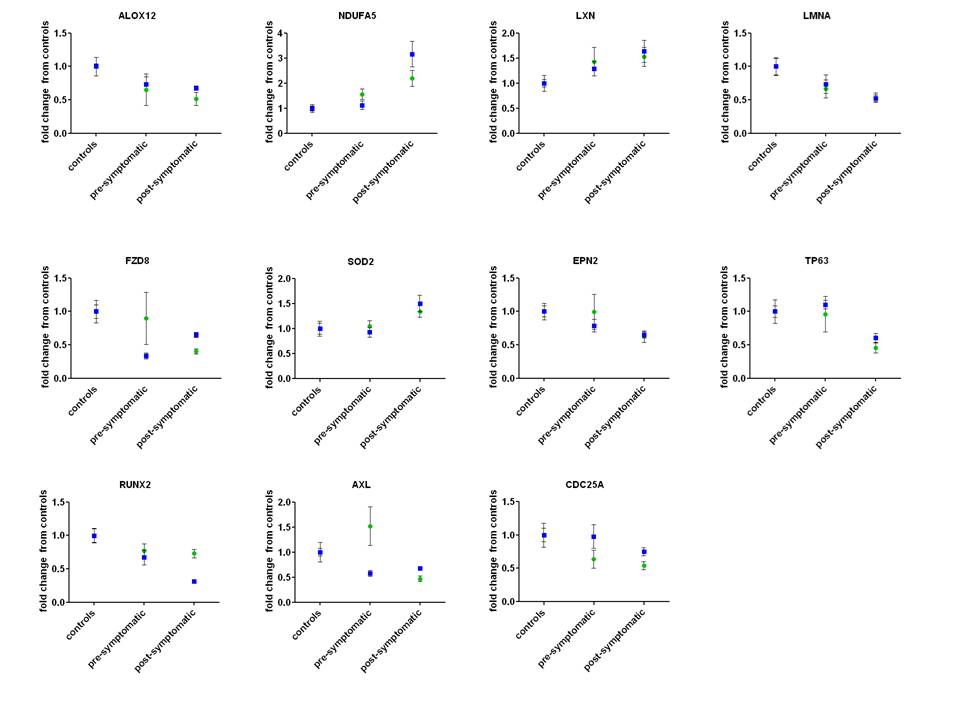

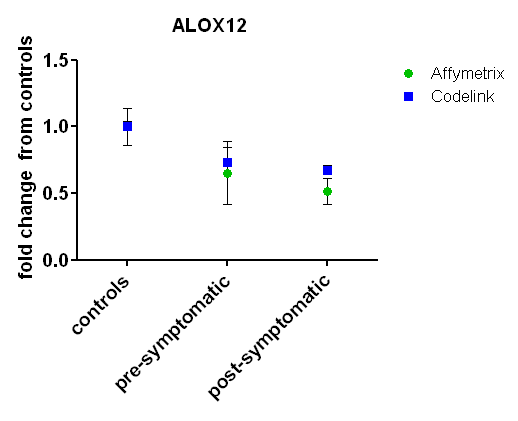


**Additional file 5. Graphs of mRNA candidates from human HD blood.** Array data deposited into the GEO database (GDS 1331 and GDS1332) from human control, HD pre-symptomatic, and HD post-symptomatic blood for 11 selected transcripts with parallel disruption in the HD monkey profiling were graphed to show disease-associated trends. All of the represented human transcripts have a P value < 0.05 for control versus post-symptomatic samples.
