# Supplementary material for: Longitudinal transcriptomic dysregulation in the peripheral blood of transgenic Huntington’s disease monkeys
Source: BMC Neurosci. 2013 Aug 17;14:88. doi: 10.1186/1471-2202-14-88 (PMC3751855; doi:10.1186/1471-2202-14-88)
Supplement: Additional file 7 — qPCR expression analysis of selected HD mRNA candidates at extended timepoints. The mRNA expression of eleven candidates with HD association in monkey and human blood were analyzed at additional timepoints of 29, 32, and 39 months. [file 1471-2202-14-88-S7.doc]

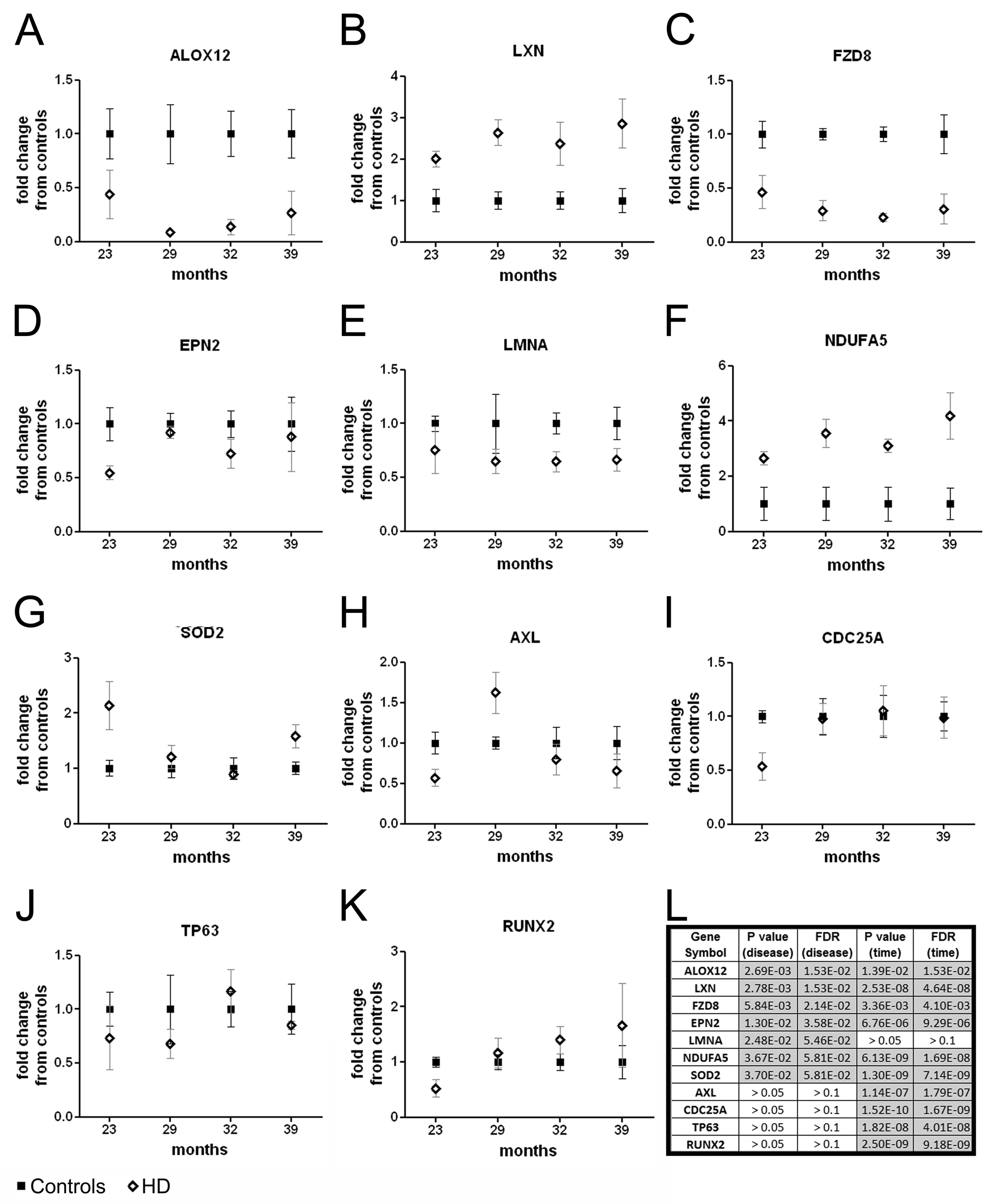


**Additional file 7.** **qPCR expression analysis of selected HD mRNA candidates at extended timepoints.** All eleven mRNA candidates identified through the microarray analysis were subjected to qPCR quantitation at 23 months as well as 3 additional timepoints of 29, 32, and 39 months. Results are shown as fold change and statistical analysis by Two-Way ANOVA for all candidates is indicated in panel L.
