# Supplementary material for: Longitudinal transcriptomic dysregulation in the peripheral blood of transgenic Huntington’s disease monkeys
Source: BMC Neurosci. 2013 Aug 17;14:88. doi: 10.1186/1471-2202-14-88 (PMC3751855; doi:10.1186/1471-2202-14-88)
Supplement: Additional file 8 — HD Rating Scale. Parameters measured in the HD primate rating scale. [file 1471-2202-14-88-S8.docx]

**Additional file 8. Huntington’s Disease Primate Motor Rating Score (HDPMRS)**

|  | | | **Score** |
| --- | --- | --- | --- |
| Motor ability | | |  |
| Bradykinesia | | |  |
| Rigidity | Upper extremity | Left |  |
|  |  | Right |  |
|  | Upper extremity | Left |  |
|  |  | Right |  |
|  | Neck |  |  |
|  | Tail |  |  |
| Dystonia | Upper extremity | Left |  |
|  |  | Right |  |
|  | Upper extremity | Left |  |
|  |  | Right |  |
|  | Neck |  |  |
|  | Trunk |  |  |
| Chorea | Upper extremity | Left |  |
|  |  | Right |  |
|  | Upper extremity | Left |  |
|  |  | Right |  |
|  | Trunk |  |  |
|  | Face |  |  |
| Diagnosis confidence level |  |  |  |
| **Total score** | | |  |

**Motor assessment**

**Motor ability**: The quality and ability of locomotion is assessed. Scale is:

“0”=normal (walking and climbing without difficulty)

“1”=walking and limited ability to climb (only able to climb for a short time span/ limited height)

“2”= walking but not unable to climb

“3”=walking with difficulty (possess a weak ability to walk)

“4”=none (unable walk or stand)

**Bradykinesia**: means slow moving and impaired ability to adjust the body’s position. Scale is:

“0”=normal (movements occur normally, without hesitation)

“1”=minimally slow (movement is minimally slow, slightly slower than normal)

“2”= mild but clearly slow (movement is undoubtedly slower than normal)

“3”= moderately slow with some hesitation (movement is slow with slight hesitation, lasting less than a couple seconds)

“4”= markedly slow and long delays in hesitation (movement are clearly slow and the animal hesitates for more than 2 seconds)

**Rigidity**: the examiner rates the stiffness/tenseness of muscles in the arms, legs, neck and tail. Scale is:

“0”=absent (limbs can easily be bent and no stiffness is observed)

“1”=slight (muscles exhibit a slight amount of tension)

“2”=mild or moderate (muscles are moderately tense)

“3”=severe with full range of motion (muscles are extremely tense however they are still capable of full range of movement)

“4”= severe with limited range (muscles are extremely rigid and are unable to move with full range)

**Dystonia**: the frequency and severity of dystonia is assessed. Dystonia is described as disordered tonicity of muscles which results in prolonged involuntary muscle contractions leading to twisting body motions, tremors and abnormal posture. Dystonia can involve the entire body or an isolated area, thus the parts of the body are addressed independently. Scale is:

“0”=absent (nothing resembling dystonia is observed)

“1”=slight/intermittent (slight expression observed occasionally)

“2”=mild/common or moderate/intermittent (slight expression frequently or moderate expression observed occasionally)

“3”=moderate/common (moderate expression observed frequently)

“4”=marked/prolonged (definite expression observed continuously)

**Chorea:** the frequency and severity of chorea is assessed. Chorea is characterized by brief irregular contractions that are not repetitive or rhythmic but nevertheless appear to flow from one muscle to the next. Chorea is also rated in individual areas of the body. Scale is:

“0”=absent (nothing resembling chorea is observed)

“1”=slight/intermittent (slight expression observed occasionally)

“2”=mild/common or moderate/intermittent (slight expression frequently or moderate expression observed occasionally)

“3”=moderate/common (moderate expression observed frequently)

“4”=marked/prolonged (definite expression observed continuously)

**Diagnosis confidence level**: The examiner rates their confidence in which the subject’s motor symptoms, if any, are related to the onset and presence of HD. Scale is:

“0”=normal (no abnormalities)

“1”=non-specific motor abnormalities (less than 50% confidence)

“2”=motor abnormalities that may be signs of HD (50-89% confidence)

“3”=motor abnormalities that are likely signs of HD (90-98% confidence)

“4”=motor abnormalities that are unequivocal
