# Supplementary material for: Longitudinal transcriptomic dysregulation in the peripheral blood of transgenic Huntington’s disease monkeys
Source: BMC Neurosci. 2013 Aug 17;14:88. doi: 10.1186/1471-2202-14-88 (PMC3751855; doi:10.1186/1471-2202-14-88)
Supplement: Additional file 10 — Microarray methods. Additional information regarding the Rhesus Macaque Affymetrix profiling are outlined. [file 1471-2202-14-88-S10.doc]

## Additional file 10

# Microarray

Rhesus lymphocytes RNA samples were sent to Ocean Ridge Biosciences (ORB, Palm Beach Gardens, FL) for analysis using Rhesus Macaque Genome GeneChips. Rhesus Macaque Genome GeneChips were purchased from Affymetrix, Inc. (Santa Clara, CA) and contained over 52,000 probesets to interrogate the known genes in the rhesus genome. For more information about the Rhesus Macaque Genome GeneChips, please refer to Affymetrix website, (<http://media.affymetrix.com/support/downloads/package_inserts/rhesus_insert.pdf>)

# Sample Processing

Quality of the total RNA was assessed using UV spectrophotometry and agarose gel electrophoresis. The total RNA was treated with DNase I (Epicentre Biotechnologies, Madison, WI) to remove any trace of DNA. Biotin-labeled complementary RNA (aRNA) was prepared from DNase treated total RNA by using the Affymetrix GeneChip IVT Express Kit (PN 901229). Briefly total RNA was reverse transcribed to generate cDNA, followed by second strand synthesis to generate a double stranded cDNA. The double stranded cDNA was then used as a template to synthesize aRNA by in-vitro transcription and incorporates a biotin-conjugated nucleotide. The aRNA was then purified to remove unincorporated NTPs, salts, enzymes, and inorganic phosphate. Quality of the labeled aRNA was assessed by 1.5 % agarose - 2% formaldehyde gel electrophoresis. The biotin-labeled aRNA was then fragmented prior to hybridization onto Rhesus Macaque Genome GeneChips. The GeneChips were hybridized for 16-18 hours under constant rotation in a GeneChip Hybridization Oven Model 640. After hybridization, the GeneChips were stained and washed under stringent conditions using the GeneChip Fluidics Station Model 450 and scanned using GeneChip Scanner 3000 Model 7G Plus as recommended in the GeneChip Expression Analysis Technical Manual (PN 702232) (Affymetrix, Inc., Santa Clara, CA).

**Data Pre-Processing**

Scanned images (CEL files) were analyzed using MAS 5.0 & RMA algorithms to export the data CHP files using the Affymetrix Expression Console software version 1.1. Data for 1,163,030 probes on the GeneChip were adjusted for background, quantile normalized, summarized and log2-transformed by the Expression Console to obtain 52,865 probeset intensities. Data was filtered for rhesus non-control probesets to obtain 52,024 rhesus probesets for a total of 32 GeneChips. The rhesus probeset intensities were further filtered to identify all 32,617 probesets detected in at least 10% of the samples based on the detection calls made by the MAS 5.0 algorithm.

**Microarray Quality Control**

The log2-transformed and normalized quality control probesets were filtered for Bacterial poly-A controls, Hybridization controls. The signal distribution and signal correlation between samples were also examined and presented as the Quality Control report.

# Differential Expression Analysis

For statistical analysis, samples were binned into two treatment groups (HD, CTRL) and four different time points (5,11,17,23 months). The log2-transformed and normalized rhesus probeset intensities for all 32,617 probesets were examined for differences between the treatment groups and also between the time points by 2-way ANOVA using BRB-ArrayTools, version 4.1.0, Beta_3 release developed by Dr. Richard Simon, National Cancer Institute. The ANOVA was conducted using the fixed effect model with two factors. The statistical significance was determined using the False Discovery Rate (FDR) method which was proposed by Benjamini and Hochberg **(1)**. It is the proportion of false positives among all probes with P values lower or equal to the P value of the probes that we consider significant. It can also be viewed as an equivalent of a P-value in experiments with multiple hypotheses testing. FDR is an intermediate method between the P-value and Bonferroni correction (multiplying P-value by the total number of probes). The equation is:


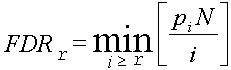


where r is the rank of a probe ordered by increasing P values, p_i_ is the P value for probe with rank i, and N is the total number of probes tested. FDR value increases monotonously with increasing P value.

ANOVA using National Institute of Ageing (NIA) Array Analysis software **(2)** was also performed on the detectable 32,617 probe sets to examine for differences between the treatment groups (HD, CTRL). Principal Component Analysis was performed on rhesus probe sets that were significant (FDR < 0.05) between the treatment groups using the module built in to the NIA software.

# Hierarchical Clustering Of Gene Expression Data

Log2 transformed and normalized data for the significant 3,133 rhesus probesets (FDR < 0.05) complementary to both coding and non-coding mRNA were clustered using Cluster 3.0 software **(3)**. The data was pre-processed by three consecutive rounds of gene median centering. Data was then hierarchically clustered using centered correlation as the similarity metric and average linkage as clustering method. Intensity scale shown is arbitrary.

# References

1. Benjamini, Y. & Hochberg, Y., (1995). J Roy Stat Soc B 57: 289-300
2. Sharov, A.A., Dudekula, D.B., Ko, M.S.H. (2005) Principal component and significance analysis of microarrays with NIA Array Analysis tool. Bioinformatics. 21(10): 2548-9.
3. De Hoon, M. J. L., Imoto, S., Nolan, J. Nolan, and Miyano, S. (2004) Open Source Clustering Software. Bioinformatics, 20 (9): 1453-1454.
